# Transcriptome and co-expression network revealed molecular mechanism underlying selenium response of foxtail millet (*Setaria italica*)

**DOI:** 10.3389/fpls.2024.1355518

**Published:** 2024-03-11

**Authors:** Yinyuan Wen, Liuna Cheng, Zeya Zhao, Mengyao An, Shixue Zhou, Juan Zhao, Shuqi Dong, Xiangyang Yuan, Meiqiang Yin

**Affiliations:** ^1^ College of Agronomy, Shanxi Agricultural University, Jinzhong, China; ^2^ Ministerial and Provincial Co-Innovation Centre for Endemic Crops Production with High-quality and Effciency in Loess Plateau, Jinzhong, China

**Keywords:** foxtail millet, Selenium biofortification, RNA-sequencing, WGCNA, sulfate transporters, phytohormones

## Abstract

**Introduction:**

Selenium-enriched foxtail millet (*Setaria italica*) represents a functional cereal with significant health benefits for humans. This study endeavors to examine the impact of foliar application of sodium selenite (Na_2_SeO_4_) on foxtail millet, specifically focusing on selenium (Se) accumulation and transportation within various plant tissues.

**Methods:**

To unravel the molecular mechanisms governing selenium accumulation and transportation in foxtail millet, we conducted a comprehensive analysis of selenium content and transcriptome responses in foxtail millet spikelets across different days (3, 5, 7, and 12) under Na_2_SeO_4_ treatment (200 μmol/L).

**Results:**

Foxtail millet subjected to selenium fertilizer exhibited significantly elevated selenium levels in each tissue compared to the untreated control. Selenate was observed to be transported and accumulated sequentially in the leaf, stem, and spikes. Transcriptome analysis unveiled a substantial upregulation in the transcription levels of genes associated with selenium metabolism and transport, including sulfate, phosphate, and nitrate transporters, ABC transporters, antioxidants, phytohormone signaling, and transcription factors. These genes demonstrated intricate interactions, both synergistic and antagonistic, forming a complex network that regulated selenate transport mechanisms. Gene co-expression network analysis highlighted three transcription factors in the tan module and three transporters in the turquoise module that significantly correlated with selenium accumulation and transportation. Expression of sulfate transporters (SiSULTR1.2b and SiSULTR3.1a), phosphate transporter (PHT1.3), nitrate transporter 1 (NRT1.1B), glutathione S-transferase genes (GSTs), and ABC transporter (ABCC13) increased with SeO_4_^2-^ accumulation. Transcription factors MYB, WRKY, and bHLH were also identified as players in selenium accumulation.

**Conclusion:**

This study provides preliminary insights into the mechanisms of selenium accumulation and transportation in foxtail millet. The findings hold theoretical significance for the cultivation of selenium-enriched foxtail millet.

## 1 Introduction

Selenium (Se), an essential trace element in the human body (Kieliszek, 2019), is crucial for forming the active site of glutathione peroxidase as selenocysteine. Its nutritional and health benefits include antioxidant, anti-tumor, anti-aging, radiation protection, antiviral effects, visual protection, and immune enhancement (Bjørklund et al., 2022), earning it the moniker “king of anticancer among trace elements” among trace elements in the human body. Se deficiency may result in various diseases such as Kaschin–Beck disease, chronic degenerative diseases, and skeletal muscle myopathy, potentially contributing to cancer and immune dysfunction (Zhang et al., 2022). A belt of Se deficiency exists in the Northern and Southern Hemispheres, involving over 40 countries and approximately one billion people, particularly in China, Africa, India, and Eastern Europe (Gao et al., 2011). As the human body cannot synthesize Se, dietary supplementation is the safest way to meet Se requirements (Rider et al., 2010). Utilizing biofortification techniques to enhance the nutritional value of staple crops is a cost-effective and feasible approach to mitigate micronutrient deficiencies (Ingle et al., 2023). Foxtail millet, rich in carbohydrates, proteins, fatty acids, vitamins, and minerals, is considered one of the most important nutritional cereals (Xiang et al., 2019), and its biofortification significantly contributes to nutritional security (Kaur et al., 2019). Biofortification with Se, effectively increasing the Se content of edible crops, has gained attention. Compared to soil application, foliar Se biofortification is more efficient and environmentally friendly, easily absorbed through leaves, and accumulates in the plant (Gao et al., 2023).

As a result of the chemical analogy of selanate/selenite with sulphate and phosphate, their behavior in metabolism and transport in plants is closely related (Raina et al., 2021). selenite may be transported through phosphate transporters and selenate through sulfate transporters (Mushtaq et al., 2022). Plants primarily absorb selenate (SeO_4_^2−^) or selenite (SeO_3_^2−^) through specific or non-specific Se transportation proteins, but not insoluble elemental Se (Se^0^) or metal selenides (White and Broadley, 2009). Selenium is subsequently transformed into organic forms such as selenocysteine, selenomethionine, and other methylated derivatives (Holben and Smith, 1999). SeO_4_^2-^ uptake in higher plants mainly occurs via sulfate transport, which is incorporated into the plant through the sulfur assimilation pathway (El Mehdawi et al., 2018). The complex mechanism of SeO_3_^2−^ accumulation and transportation in plants remains unclear. Phosphate transporters (OsPHT1.2 and OsPHT1.8) and the aquaporin NIP2;1 in rice participate in SeO_3_^2−^ accumulation and transportation (Li et al., 2008; Zhao et al., 2010; Zhang et al., 2014). The nitrate transporter (NRT1.1B) promotes the transport of selenomethionine (SeMet) in rice (Zhang et al., 2019). ABC transporters may also be involved in Se absorption and transport in plants. ABCC11, ABCC13, and ABCC10 are implicated in the accumulation and transportation of nanoselenium in cowpeas, regulating Se absorption and transformation (Li et al., 2023). Studies indicate that Se may regulate the expression levels of GSTs, affecting transcription factor activity or participating in signal transduction pathways (Zheng et al., 2023).

Compared with Arabidopsis and rice, research on the mechanism of Se transportation in foxtail millet is limited. This study aimed to investigate the effects of foliar spraying of sodium selenite (Na_2_SeO_4_) on foxtail millet (Setaria italica) concerning selenium (Se) accumulation and transportation within different plant tissues. Specific focus was given to the dynamics of Se content, RNA expression patterns, identification of differentially expressed genes (DEGs), functional annotations related to Se transport, and the role of various transporters, hormones, antioxidants, and transcription factors in Se accumulation. A detailed RNA-Seq analysis of the head stage of foxtail millet using selenium and water sprays was conducted, alongside the measurement of Se content in each foxtail millet tissue. These data offer a comprehensive system-level view of dynamic gene expression networks and their potential roles in Se accumulation and transportation. Using pairwise comparisons and weighted gene co-expression network analysis (WGCNA), candidate hub gene modules were identified. Through WGCNA, co-expressed gene modules were constructed, and a correlation analysis with selenium content data identified key modules related to selenium accumulation and transportation. Hub genes within these modules associated with Se accumulation and transportation were subsequently identified.

## 2 Materials and methods

### 2.1 Plant materials and treatments

Jingu 21 foxtail millet served as the test material. Field experiments were conducted from May to October 2022 at the Shanxi Agricultural University experimental station (Shenfeng Village, Taigu County, Jinzhong City, Shanxi Province, China). The experiment was performed using a completely randomized design with three replicates. Square plots, 25 m^2^ in size, were used with 35-cm row spacing and 8 cm plant spacing. Nitrogen (150 kg·hm^−2^ of N), phosphorus (90 kg·hm^−2^ of P_2_O_5_), and Potassium (120 kg·hm^−2^ K_2_O) fertilizers were applied before sowing. Local production recommendations were used in management of crops in the field. During the heading stage, we applied a foliar spray of 200 μmol/L Na_2_SeO_4_ for selenium treatment, while the control group received an equivalent volume of water (75 mL/m^2^). Whole healthy roots, stems, functional leaves, stalks of spikelets, and spikes, along with seeds at the filling stage (S1-S5) (He et al., 2022), were collected on the 3rd, 5th, 7th, and 12th days of treatment. Each treatment comprised three biological replicates. For transcriptome analysis, spikes were collected, swiftly frozen in liquid nitrogen, and preserved at -80°C for subsequent physiological evaluations, RNA extraction, and gene expression analysis.

### 2.2 Foxtail millet selenium content measurement

We measured approximately 0.3 g of the sample (accurate to 0.0001 g) and placed it in a digestion tube. Subsequently, we introduced 6 mL of nitric acid and 2 mL of hydrogen peroxide into the tube, which was then sealed using a microwave digestion instrument. The digestion process involved heating to 120°C for 10 min, followed by heating to 150°C for another 10 min, and finally heating to 180°C for 30 min. After cooling, we added 5 mL of hydrochloric acid solution (6 mol/L). The tube was then opened and placed in a fume hood, and acid evaporation occurred at 170°C until 2 mL of liquid remained. Following digestion and cooling to ambient temperature, the samples were diluted with ultrapure water to a final volume of 10 mL and subjected to shaking. The Se content in various tissues was determined using inductively coupled plasma mass spectrometry (ICP-MS).

### 2.3 RNA sample collection and illumina sequencing

Total RNA samples from the spikes of JG21 under water and selenium treatments were extracted using RNAprep Pure reagent (QIAGEN, Germany) following the manufacturer’s protocol. Illumina NovaSeq 6000 Sequencer at Beijing Novogene Biotechnologies Company, Beijing, China, was used for RNA-Seq. After filtering, clean sequence read segments were compared to the *Setaria_italica*_v2.0 reference genome using HISAT v.2.0.5. HTSeq was used to estimate the number of base fragments per kilobase of transcripts per million mapping reads (FPKM). Principal component analysis (PCA) was performed using log2 (FPKM+1) transformation and normalized gene expression values with the fast. Prcomp function from the models in R version 3.5.1.

### 2.4 DEG identification and functional analyses

DEG identification and functional analysis were conducted using DESeq v.1.20.0. In each pairwise comparison, DEGs were identified with a Benjamini and Hochberg false discovery rate (FDR) < 0.05, FPKM > 1, and |log2 fold change (FC)| > 0.5. Further analyses of DEGs, including Gene Ontology (GO) enrichment analysis, Clusters of Orthologous Groups of Proteins (COG) analysis, Kyoto Encyclopedia of Genes and Genomes (KEGG) analysis, and NCBI nonredundant protein sequence (Nr) annotation, were performed.

### 2.5 WGCNA

Gene co-expression modules were constructed using the R package WGCNA v3.5.0. To identify Se accumulation in the Se- and CK-related modules under treatment, we correlated the eigengene module with Se content and drew their correlation heat maps. Genes with an average FPKM > 1 out of 24 samples were analyzed. The soft threshold power β was set at five, and mergeCutHeight = 0.4 was used to merge similar modules. If the p-value of the module-trait association is 0.05, then the module is defined as significant (Wang et al., 2022). The OmicShare tool2 (https://www.omicshare.com/) was used to map the network visualization of genes within the module. Genes with high co-expression connectivity within the screening module were visualized using Cytoscape v.3.7.2 (Seattle, WA, USA).

### 2.6 qRT-PCR analysis

For the synthesis of first-strand cDNA, 0.5 μg of purified RNA underwent reverse transcription using the Takara PrimeScript RT Reagent Kit (TaKaRa, Beijing, China), including gDNA Erase, following the manufacturer’s instructions. Subsequently, qRT-PCR was performed on a CFX96 Real-Time System (Bio-Rad, Hercules, CA, USA) using Super Real Premix Plus (SYBR Green) (TaKaRa, Beijing, China). Specific primers for the 10 selected genes were designed using the Primer Premier 5.0 design tool (**Supplementary Table 1**). The relative expression level of the gene was determined using the 2^-ΔΔCt^ method, with Actin (*SETIT_004277 mg*) as the internal reference gene. Bar charts were generated using Origin 2022, and significance analysis (P < 0.05) was performed using SPSS 26.

## 3 Results

### 3.1 Selenium content in each tissue of foxtail millet

**Figure 1:**
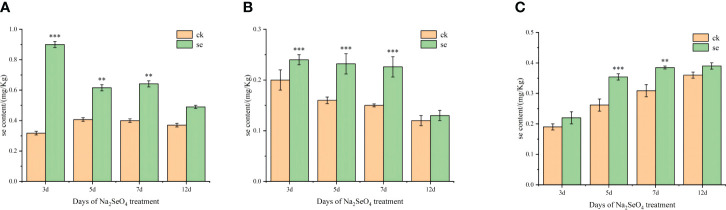
Effects of spraying sodium selenate on selenium content in leaves **(A)**, stems **(B)**, and spikes **(C)** of foxtail millet at the heading stage. *, ** and *** mean significant correlation at 0.05, 0.01 and 0.001 levels, respectively.

The Se content in every tissue of foxtail millet exhibited an increase post-Se spraying (**Figure 1**). Interestingly, the Se content in leaves gradually decreased over time, while that in spikes exhibited a gradual increase. As the treatment duration extended, Se content decreased in leaves and stems, concomitant with an increase in spikes. This pattern suggests that foliar spraying of Na_2_SeO_4_ during the heading stage facilitated the sequential transport and accumulation of selenate in leaves, stems, and spikes.

### 3.2 Quality assessment of RNA-seq data

**Figure 2:**
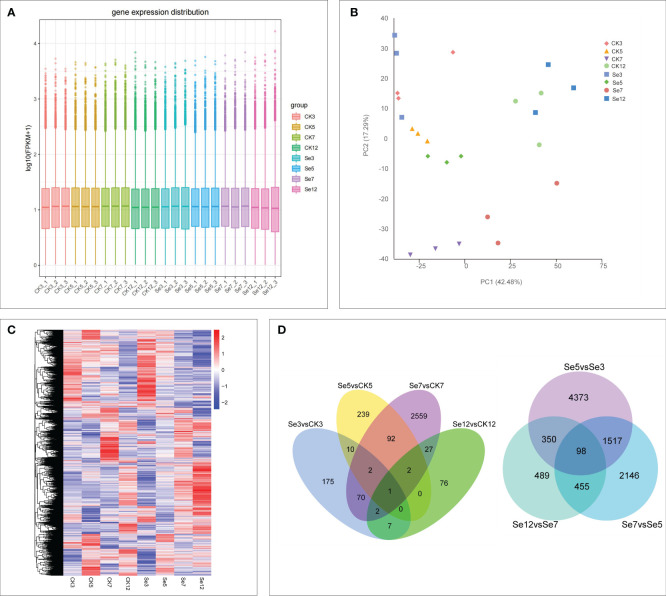
Global transcriptome sequencing and differentially expressed genes (DEGs) in CK and Se of foxtail millet. Principal component analysis (PCA) of RNA-sequencing (RNA-Seq) data **(A)**. Gene expression of all samples. The boxplots with different colors indicate different samples analyzed at regular intervals **(B)**. Cluster analysis of DEGs based on gene expression of all samples **(C)**. Venn diagrams showing the number of DEGs among seven comparisons **(D)**. CK3 (water treatment day 3), CK5 (water treatment day 5), CK7 (water treatment day 7), CK12 (water treatment day 12), Se3 (Na_2_SeO_4_ treatment day 3), Se5 (Na_2_SeO_4_ treatment day 5), Se7 (Na_2_SeO_4_ treatment day 7), Se12 (Na_2_SeO_4_ treatment day 12). The same below.

To explore the dynamic effects of Na_2_SeO_4_ on the expression of selenate transport-related genes during foxtail millet spike development, we conducted RNA-seq analysis on the spikes of JG21 plants treated with water and Na_2_SeO_4_ during the heading stage. Each sample, including CK3, Se3, CK5, Se5, CK7, Se7, CK12, and Se12, with three biological replicates, underwent quality assessment. A total of 154.03 G clean data was obtained from 24 samples, with individual samples ranging from 5.76 to 6.95 G. The Q30 value exceeded 91.26%, and the GC content distribution was 52.16-54.78% (**Supplementary Table 2**). After filtering low-quality reads, 84.38%-95.16% mapped to the *Setaria_italica*_v2.0 reference genome (**Supplementary Table 2**). PCA revealed significant differences between the eight treatments, with all replicates closely clustered. PC1 and PC2 contributed 42.48% and 17.29% to the total difference, respectively (**Figure 2A**). The results indicated varied gene expressions over time following foxtail millet water spraying and Na_2_SeO_4_ treatment, suggesting specific responses possibly linked to selenium transport in foxtail millet spikes. With high quality sampling, sequencing, and gene quantification, we identified differential genes associated with selenium transportation in foxtail millet spikes.

### 3.3 DEGs analysis and functional annotations

Evaluation of FPKM values depicted the expression of all genes (**Figure 2B**). Transcript abundance comparisons across samples led to the identification of differentially expressed genes (DEGs) in each sample (**Figures 2C, D**), revealing increased sensitivity of gene expression in foxtail millet spikes to Na_2_SeO_4_ treatment. Notably, comparing Na_2_SeO_4_ treatment with water treatment unveiled 3,262 unique DEGs across four comparisons (**Figure 2D**). Comparing 9,428 unique DEGs under Na_2_SeO_4_ treatment on adjacent days (days 3, 5, 7, and 12) revealed 98 common DEGs in the three comparisons (**Figure 2D**). These results strongly suggest that Na_2_SeO_4_ exerts regulatory control over the expression of a substantial number of genes.

For deeper insights into the potential mechanisms underlying selenium transport in foxtail millet spikes, functional classification of DEGs from all seven comparisons was conducted using GO enrichment analysis. Key terms included “thylakoid,” “thylakoid membrane,” “stroma,” and “photosynthetic membrane” under cellular components; “binding,” “transporter activity,” and “transferase activity” under molecular function, and “cellular processes,” “metabolic processes,” and “response to stimulus” under biological processes (**Supplementary Table 3**). Additionally, KEGG pathway analysis among the seven comparisons highlighted critical processes such as “selenocompound metabolism (map00450),” “plant hormone signal transduction (map04075),” “glutathion metabolism (map00480),” and “ABC transporters (map02010)” (**Supplementary Figure 1**). These findings illuminate the crucial biochemical pathways and genes regulating selenium accumulation after Se spraying on foxtail millet leaves, offering insights for the development of functional Se-enriched millet varieties. Further investigations are warranted to explore the DEGs involved in these pathways.

### 3.4 DEGs involved in selenium metabolism and transportation

**Figure 3:**
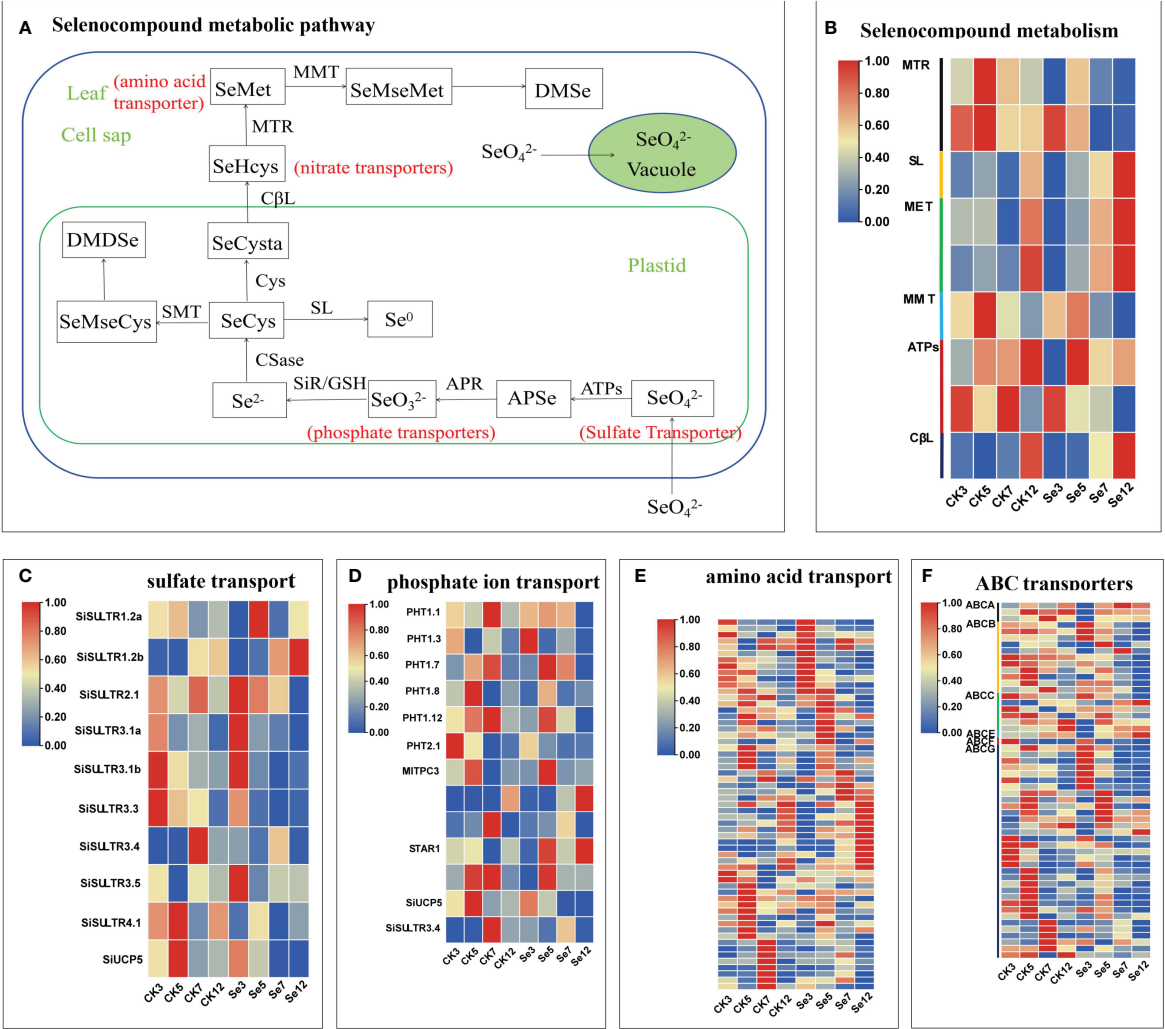
Selenocompound metabolism **(A)**. Expression profiles of differentially expressed genes (DEGs) involved in selenocompound metabolism **(B)**, sulfate transport **(C)**, phosphate transport **(D)**, amino acid transport **(E)**, and ABC transporter **(F)**. Different colors indicate different gene expression levels based on log2 FoldChange. The same below.

To unravel the molecular intricacies governing selenium (Se) metabolism and transportation in millets, we pinpointed DEGs associated with selenocompound metabolism and sulfate transporters. In nature, Se manifests in organic and inorganic forms, further categorized based on its oxidation state as elemental selenium (Se^0^), selenide (Se^2-^), selenite (Se^4+^), and selenate (Se^6+^) (White and Broadley, 2009). Selenate, upon entering the chloroplast, undergoes activation by ATP sulfurylase (ATPs) to generate 5’-adenosine phosphoselenate (APSe). Subsequently, 5’-adenosine phosphosulfate reductase (APR) catalyzes APSe to form selenite. Notably, both externally absorbed selenate and selenite traverse the same assimilation pathway (Schiavon et al., 2015) (**Figure 3A**). Concurrently, sulfate transporters contribute to SeO_4_^2−^ accumulation (Zou et al., 2021), phosphate transporters facilitate SeO_3_^2−^ accumulation (Zhang et al., 2014), amino acid transporters engage in selenide metabolism (Taylor et al., 2015), and nitrate transporter promotes selenomethionine (SeMet) transport (Zhang et al., 2019). Additionally, the responses of ABC transporters to Se metabolism and transportation were explored.

Following the treatment of foxtail millet spikes with CK and Se on different days, we identified nine unique DEGs linked to the selenocompound metabolic pathway in five comparisons (**Figure 3B**; **Supplementary Table 4**). Moreover, 10 unique DEGs associated with sulfate transporters emerged from seven comparisons (**Figure 3C**; **Supplementary Table 5**), along with 13 unique DEGs related to phosphate transporters, 59 unique DEGs related to amino acid transporters, and 55 unique DEGs related to ABC transporters in seven comparisons (**Figures 3D–F**; **Supplementary Tables 5**, **6**). Notable genes, including *SiSULTR1.2a*, *SiSULTR1.2b*, *SiSULTR2.1*, *SiSULTR3.1a*, *SiSULTR3.5*, and *PHO1-3*, exhibited increased expression post-Se spraying. *SiSULTR3.4* demonstrated involvement in both sulfate and phosphate transporters. The ABC transporter family prominently featured three subfamilies: ABCB, ABCC, and ABCG, underscoring their pivotal role in the ABC transporter family’s response to Se stress in foxtail millet.

### 3.5 DEGs associated with plant hormone signal transduction

**Figure 4:**
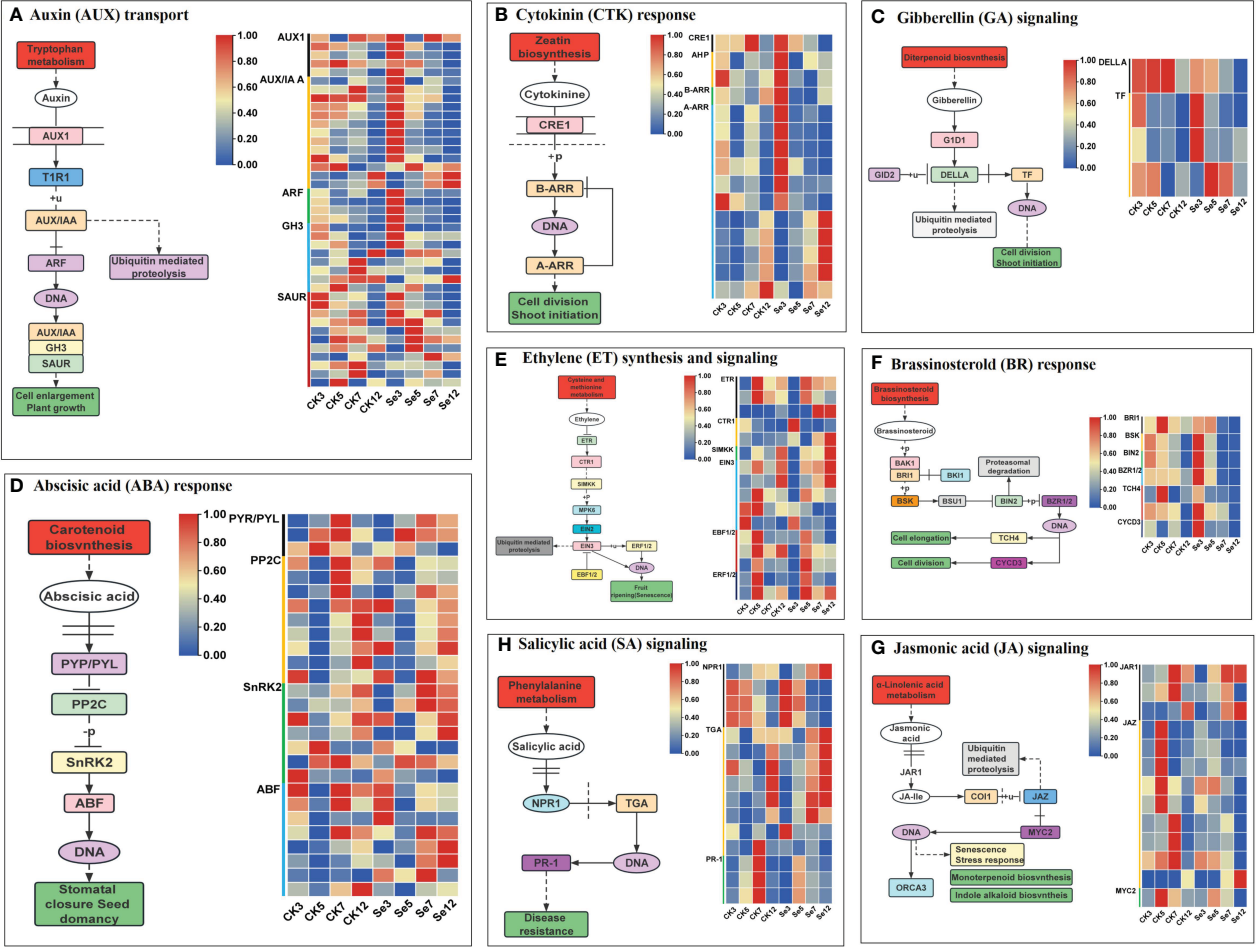
Eight plant hormone signal transduction pathways and expression profiles of differentially expressed genes (DEGs) involved in auxin (AUX; **A**), cytokinin (CTK; **B**), gibberellin (GA; **C**), abscisic acid (ABA; **D**), ethylene (ETH; **E**); brassinosteroid (BR; **F**), jasmonic acid (JA; **G**), and salicylic acid (SA; **H**) signal pathways of foxtail millet spikes after selenium and water spraying treatments.

To scrutinize Se’s impact on phytohormone signal transduction in foxtail millet, we delved into gene expression profiles within phytohormone signal transduction pathways. In the auxin pathway, Se upregulated the expression of key genes like auxin influx carrier *AUXIN1* (*AUX1*), *Auxin/indoleacetic acid* (*AUX/IAA*), and *Auxin response factor* (*ARF*) genes (**Figure 4A**; **Supplementary Table 7**). Cytokinin pathway analysis revealed Se-induced upregulation of most *Type A Arabidopsis response regulator* (*A-ARR*) genes (**Figure 4B**; **Supplementary Table 8**). Similarly, Se influenced the gibberellin pathway by upregulating one *DELLA protein* (*DELLA*) and three *phytochrome-interacting factor* (*TF*) genes (**Figure 4C**; **Supplementary Table 9**). The abscisic acid (ABA) pathway exhibited regulation by 27 unique DEGs (**Figure 4D**; **Supplementary Table 10**). Se, also modulated the ethylene pathway by downregulating certain components while upregulating others (**Figure 4E**; **Supplementary Table 11**). In the brassinosteroid pathway, Se exerted differential regulation on various genes (**Figure 4F**; **Supplementary Table 12**). Similarly, Se downregulated most *Jasmonate ZIM-domain* (*JAZ*) genes in the jasmonic acid pathway (**Figure 4G**; **Supplementary Table 13**). The salicylic acid pathway demonstrated a nuanced response with both upregulation and downregulation of specific genes (**Figure 4H**; **Supplementary Table 14**). Collectively, these results underscored Se’s impact on phytohormone biosynthesis and signaling pathways.

### 3.6 DEGs associated with antioxidation

**Figure 5:**
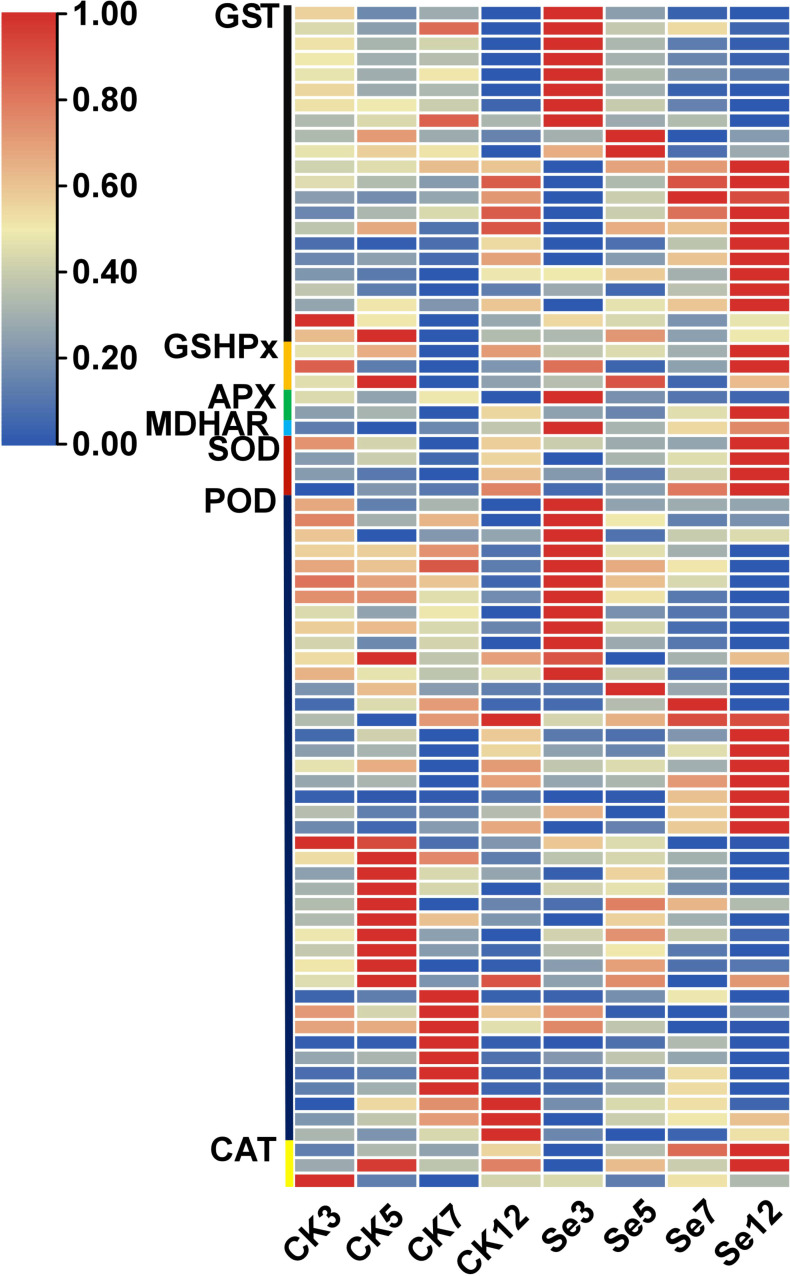
Expression profiles of differentially expressed genes (DEGs) involved in antioxidation.

The application of Se treatment significantly upregulated genes associated with the antioxidant response, including superoxide dismutase (SOD), ascorbate peroxidase (APX), catalase (CAT), peroxidase (POD), monodehydroascorbate reductase (MDHAR), glutathione peroxidase (GSH-Px), and glutathione S-transferase (GST) (**Supplementary Table 15**; **Figure 5**). Noteworthy DEGs within the antioxidant system encompassed SOD, APX, CAT, and POD, with unique expressions and regulatory patterns. Similarly, GST-related genes exhibited diverse expression dynamics, with selenium. It was identified one upregulated monodehydroascorbate reductase gene, three unique DEGs associated with the GSH-Px enzyme, and 22 unique DEGs associated with the GST enzyme, with 20 upregulated and two downregulated genes. Selenium spraying induced the early expression This response varied temporally, with certain genes showing altered expression on day 3 post-selenium treatment compared to their original expression on day 7 or 12.

### 3.7 WGCNA of foxtail millet after Se treatment

**Figure 6:**
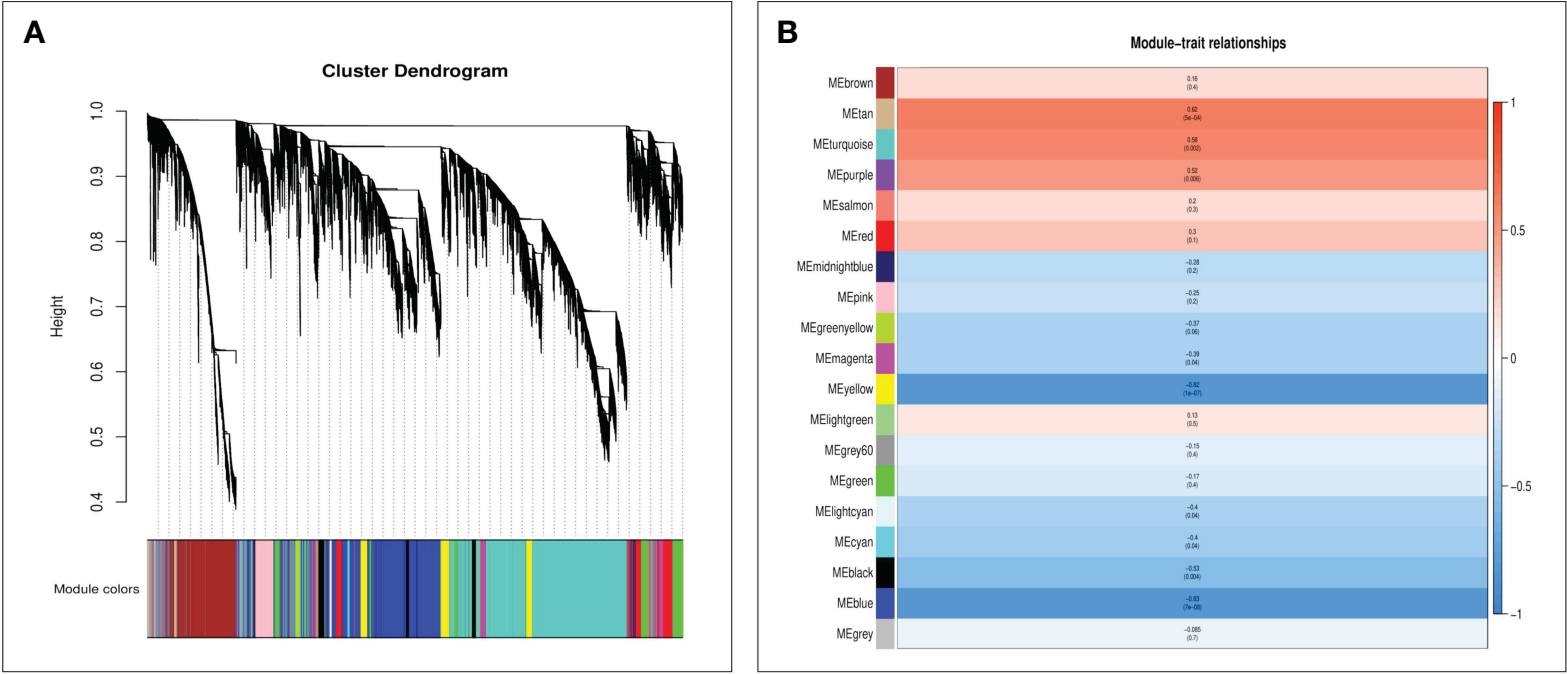
The WGCNA of foxtail millet panicle transcripts after selenium treatment used the average linkage hierarchical clustering method to construct the gene tree of CK and Se, and each row represents a gene. The module color under the clustering tree shows the result of the dynamic tree-cut module allocation **(A)**. Correlation between module characteristic genes and selenium content **(B)**. The color of each module was the same as that in **(A)**. The correlation coefficient (r) and p-value are shown in each cell.

To unravel the specifically induced regulatory network response from leaves to spikes in foxtail millet following foliar Se application, we subjected expression datasets (FPKM >1) from 24 samples to WGCNA. This analysis identified nineteen co-expression modules (mergeCutHeight = 0.40) in foxtail millet spikes (**Figure 6A**). Subsequently, we explored the correlations between these modules using the eigengene module (**Figure 6B**). Two modules, namely “tan” (r= 0.62, p=0.000) and “turquoise” (r= 0.58, p=0.002), exhibited significant positive correlations with Se content, making them the focal point of our study.

**Figure 7:**
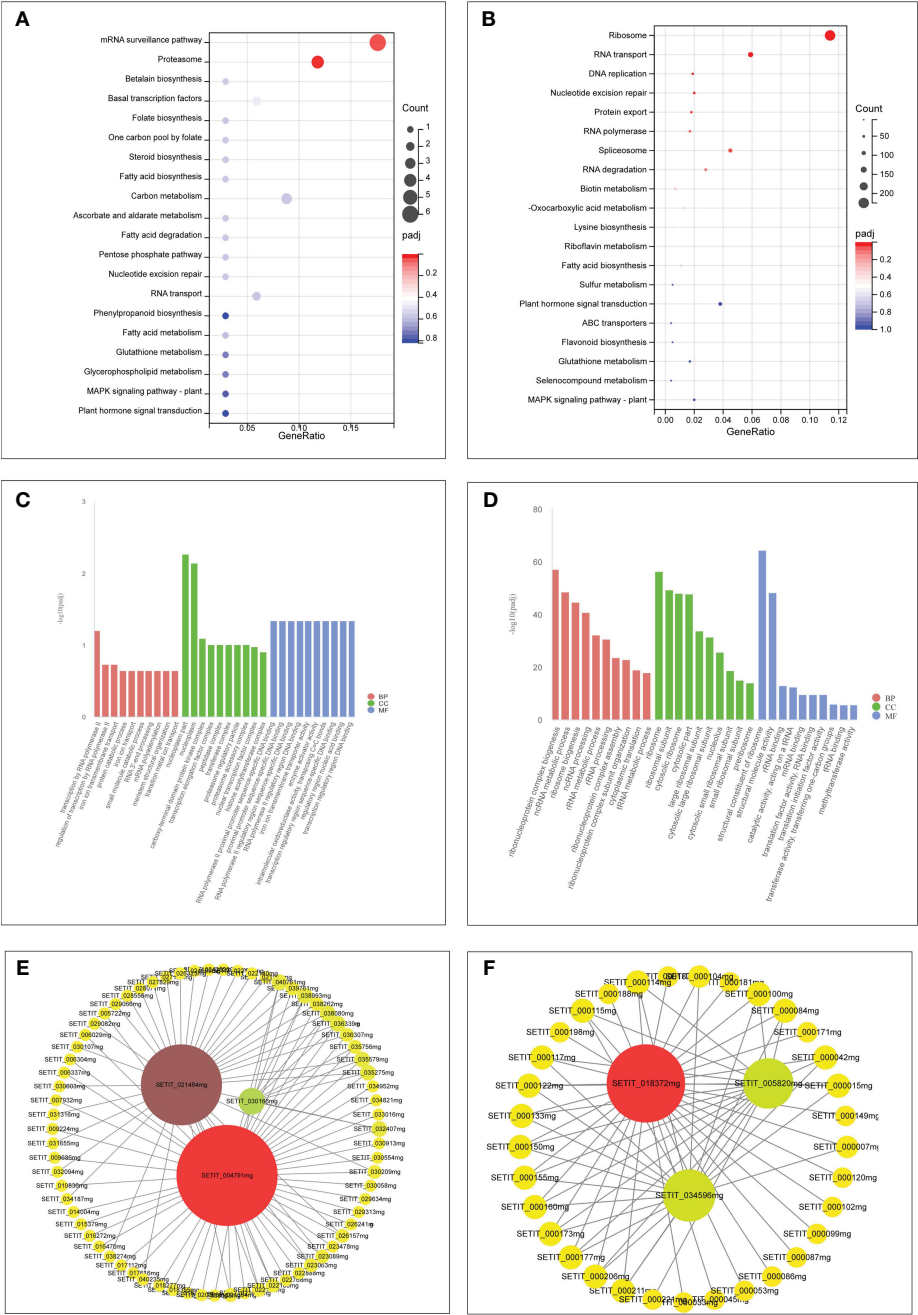
GO and KEGG analysis of unique genes in the tan module **(A, C)**. GO and KEGG analysis of unique genes in the turquoise module **(B, D)**. Hub genes network interaction in the tan module **(E)**. Hub genes network interaction in the turquoise module **(F)**.

Furthermore, GO and KEGG analyses were conducted on genes within the “tan” and “turquoise” modules to elucidate their biological functions. Both modules were enriched in GO terms related to “cellular processes,” “metabolic processes,” and “transport activities” (**Figures 7A, B**). The “tan” module demonstrated predominant enrichment in KEGG pathways such as “plant hormone signal transduction,” “Glutathione metabolism,” and “MAPK signaling pathway” (**Figure 7C**). In contrast, the “turquoise” module was enriched in pathways such as “plant hormone signal transduction,” “sulfur metabolism,” “selenocompound metabolism,” “flavonoid biosynthesis,” and “glutathione metabolism” (**Figure 7D**).

### 3.8 Identification of hub genes and interaction network in modules

Hub genes within the “tan” and “turquoise” modules, identified based on module membership >0.8 and GS>0.2, revealed three closely related genes in each module (**Figures 7E, F**). Notably, the tan module featured three transcription factors— WRKY29 (SETIT_004791 mg), MYB3R-2 (SETIT_021484 mg), and bHLH130 (SETIT_030166 mg)—all implicated in Se regulation. The turquoise module highlighted hub genes such as *SiSULTR1.2* (SETIT_034596 mg), *SiNRT2.1* (SETIT_018372 mg), and *ABCC13* (SETIT_005820 mg), associated with sulfur transport (GO: 0008272), nitrate transport (GO: 0015706), and active transmembrane transport activity (GO: 0022804), respectively.

### 3.9 Analysis of sulfate transporter protein (SULTR) expression patterns

**Figure 8:**
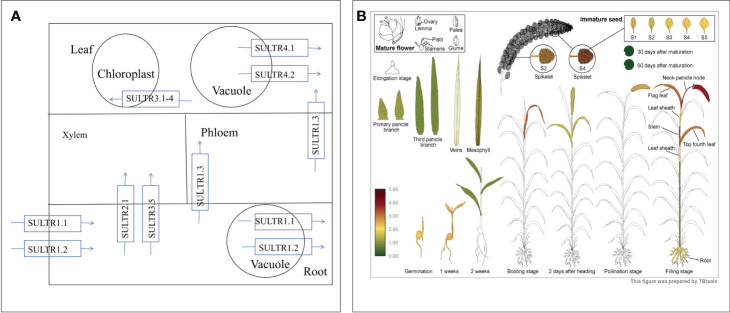
The absorption and transport mechanism of selenate by plants **(A)**. Expression of SiSULTR1.2b in different tissues of foxtail millet at different stages **(B)**.

**Figure 9:**
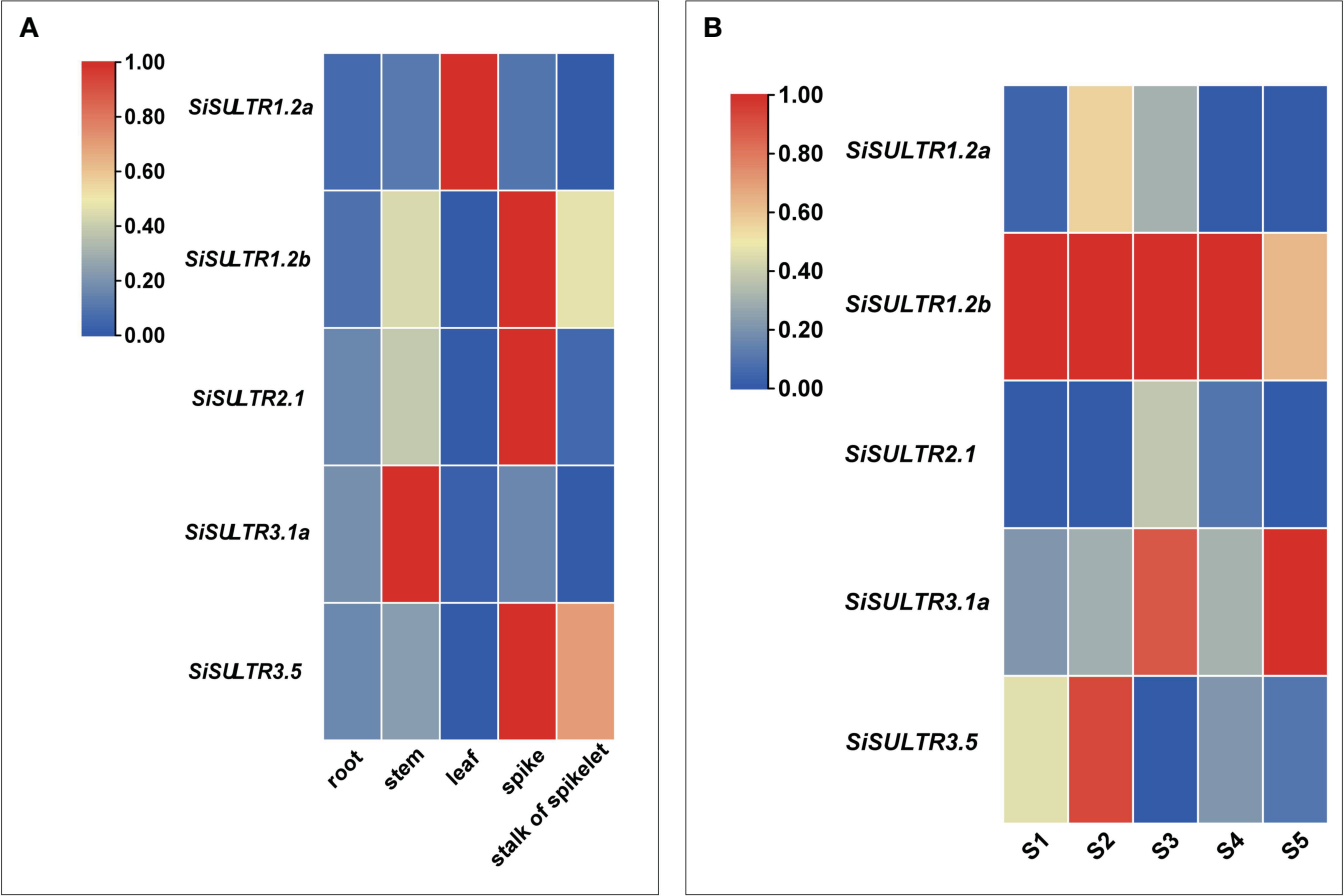
Analysis of the tissue expression pattern on the 12th day of selenium spraying **(A)**. Analysis of expression patterns in five stages of grain filling **(B)**.

Given the mediation of SeO_4_^2−^ uptake and transport by sulfate transporters, our focus turned to the sulfate transporter gene family (**Figure 8A**). Transcriptome analysis identified 10 genes encoding SULTRs (**Figure 3C**). The response patterns of *SiSULTR1.2a*, *SiSULTR1.2b*, *SiSULTR2.1*, *SiSULTR3.1a*, and *SiSULTR3.5* to selenium spraying varied temporally, with some genes responding on days 3, 7, or 12 post-treatment. Tissue-specific expression pattern analysis 12 days after selenium spraying (**Figure 9A**) and gene expression levels across five stages of grain filling (S1–S5) (**Figure 9B**) revealed *SiSULTR1.2b* as particularly notable, exhibiting high expression levels in foxtail millet spikes and maintaining consistent high expression throughout the grain -filling stages. This gene, accessed from the foxtail millet database (http://foxtail-millet.biocloud.net/home), displayed expression in grains, leaves, and roots during the grain-filling stage, with the highest expression observed in grains, suggesting a crucial role in Se transport from leaves to grains in foxtail millet (**Figure 8B**).

### 3.10 Gene expression validation through qRT-PCR

**Figure 10:**
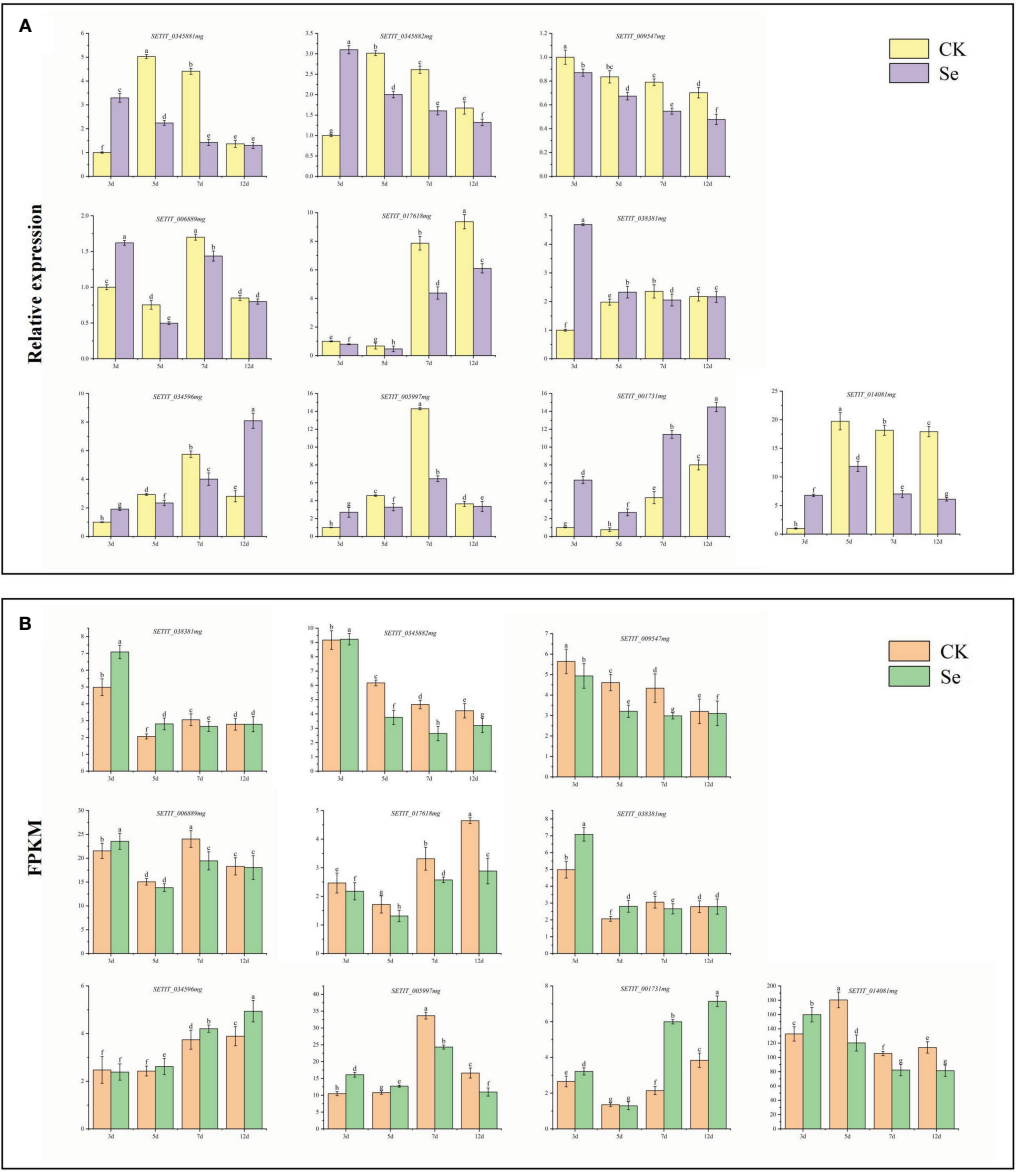
Candidate gene qRT-PCR verification **(A)**. Candidate gene RNA-Seq expression pattern **(B)**. Different lowercase letters above the bar indicate significant differences at the p < 0.05 level in the different treatment.

To validate the reliability and efficacy of the RNA-Seq data, we conducted qRT-PCR analysis on the relative expression levels of 10 selected genes, including six related to sulfur transport and four associated with plant hormone signal transduction. The qRT-PCR expression trends of JG 21 spikes (**Figure 10A**) aligned with those observed in the RNA-Seq data (**Figure 10B**), affirming the consistency between the two analytical methods.

## 4 Discussion

In this study, transcriptome analysis was employed to investigate the temporal transcriptional changes in spikes treated with selenates, elucidating the primary mechanism underlying selenium transport in foxtail millet. Transcriptomic data were utilized to comprehensively explore the enrichment pathways associated with KEGG, and GO. The principal component analysis diagram directly depicted the degree of separation between samples from different groups. The biological repeats at 3d and 5d exhibited closer proximity, whereas those at 7d and 12d displayed slightly longer distances, yet still allowing for clear classification. The gene expression distribution chart indicated a similar expression trend among samples at the heading stage. Overall, the quality control and expression validation of the omics data confirmed the reliability of our findings, thereby providing valuable support for elucidating the mechanism of selenium enrichment in millet.

### 4.1 Effects of leaf spraying selenium on the selenium content of crops

Research on grain crops has revealed a positive correlation between selenium (Se) concentration in grains and foxtail millet’s Se application. The study recorded the highest Se concentration in grains (1.83 mg/kg) when spraying 61.5 g Se hm^-2^ (Li et al., 2022). Leaf spraying of sodium selenite increased Se content and yellow pigment in foxtail millet (Ning et al., 2016). On rice grains, selenite foliar application enhanced Se concentrations in glutelin and albumin proteins, such as SeCys2 and SeMet (Hu et al., 2018). Wheat grains exhibited increased Se concentration and highly bioavailable SeMet fraction with sodium selenate foliar fertilization (Ramkissoon et al., 2019). Potatoes treated with foliar selenate showed enhanced Se concentration, attributed to improved Se fluidity in the phloem (Poggi et al., 2000). In cash crops, Se spraying during the autumn tea-producing season increased Se and vitamin C contents in green tea (Huang et al., 2005). Grape leaves treated with amino acid-chelated selenium-enriched foliar fertilizer significantly increased Se content (Yin et al., 2020). Blueberries treated with foliar selenate and selenite (200 g/ha) during the young fruit stage showed enhanced Se accumulation in the fruit (Li et al., 2018). In our study, Se content significantly increased in all foxtail millet tissues after Se spraying. Over time, Se levels in leaves decreased, while in spikes, they gradually increased, indicating Se absorption and transport to the kernels in the leaves.

### 4.2 Transporters involved in Se accumulation and transportation in foxtail millet

In the botanical realm, selenium (Se) manifests in both organic and inorganic forms. Plant-hosted inorganic selenium encompasses selenate (SeO_4_^2−^) and selenite (SeO_3_^2−^), while organic counterparts encompass selenocysteine (SeCys) and selenomethionine (SeMet) (White and Broadley, 2009). The intricate interplay of distinct absorption and transport mechanisms for varied Se forms, facilitated by specific transporters, results in the conversion of part of inorganic selenium into organic Se compounds, persisting within the plant structure. The remaining fraction undergoes metabolism, yielding volatile compounds—specifically dimethyl diselenide (DMDSe) and dimethyl selenide (DMSe) (Gui et al., 2022). SeO_4_^2−^ and SeO_3_^2−^ exhibit a robust affinity for plants. Sulfate transporters, such as SULTR1.2 in Arabidopsis, are instrumental in SeO_4_^2−^accumulation and transport (Shibagaki et al., 2002). In our investigation, nine SULTRs were identified, notably SiSULTR1.2b and SiSULTR3.1a, exhibiting upregulation during grain filling, suggesting their pivotal role in Se transport after foliar Se application (**Figures 3C**, **9B**). Phosphate transporters, participate in SeO_3_^2−^ accumulation, and hydrogen selenite (H_2_SeO_3_ and HSeO^3-^) flows through silicon influx (NIP2;1) and phosphate (PT2) transporters (Zhang et al., 2014). Phosphate transporters (OsPHT1.2 and OsPHT1.8) engage in SeO_3_^2−^ accumulation and transportation in rice (Zhang et al., 2014; Li et al., 2018). PHT1.3 in foxtail millet, play a role in accumulation, emphasizing their significance in Se transport (**Figure 3D**). The transport of Se in foxtail millet. The nitrate transporter protein NRT1.1B promotes the transport of SeMet in rice (Zhang et al., 2019). Three DEGs associated with nitrate transport were found in foxtail millet (**Supplementary Table 5**), and NRT2.1 (SETIT_ 018372mg), emerge as potential key players in Se transport in foxtail millet (**Figure 7F**).

ABC transporters, featuring a conserved ATPase domain and function by utilizing ATP to facilitate the transport of substrates across membranes. Consequently, they play a crucial role in various physiological processes, including the accumulation of plant secondary metabolites, as well as in biological and abiotic stress responses (Byrne et al., 2010). These transporters can be divided into eight subfamilies: ABCA, ABCG, and ABCI (Verrier et al., 2008). ABC transporters have been implicated in Se accumulation and transport in rice, and were detected after SeO_3_^2−^ treatment, suggesting their potential involvement in Se accumulation and transport (Kong et al., 2021). ABCC11, ABCC13, and ABCC10 engage in nano-selenium accumulation and transport in cowpeas (Li et al., 2023). ABC transporter G family member 36 in alfalfa leaves is significantly upregulated after Se treatment, suggesting its involvement in the movement of Se into the leaf tissue (Wang et al., 2021). Zheng et al. observed the upregulation of 14 ABC genes in tea trees, suggesting that they may be involved in Se accumulation and transport in tea roots (Zheng et al., 2023). In perennial ryegrass, ABCA transporters regulate Se movement and accumulation. ATH genes in the ABCA subfamily were upregulated in response to selenite exposure (Byrne et al., 2010). ABCG14 participates in phytohormone transport (Gräfe and Schmitt, 2021). In this study, six ABC subfamilies were discerned, with ABCC13 posited to play a pivotal role in Se transport from leaves to spikes in foxtail millet (**Figures 3F**, **7E**). This elucidation underscores the intricate orchestration of transporters in Se dynamics within foxtail millet, shedding light on the molecular mechanisms governing Se accumulation and transportation.

### 4.3 Plant hormone signaling pathway genes involved in Se accumulation and transportation in foxtail millet

Supplementation with Se enhances resistance to abiotic stress by modulating plant hormone homeostasis and regulating endogenous hormone levels. The generation of reactive oxygen species (ROS) in plants promotes the increase in levels of jasmonic acid and ethylene stress hormones (Overmyer et al., 2003). Se treatment triggers the expression of genes related to plant hormone signaling pathways, as demonstrated in pepper leaves treated with nano-selenium, resulting in elevated levels of jasmonic, abscisic, and salicylic acids (Li et al., 2020). The assimilation of Se is promoted by Jasmonic acid, gibberellin, and abscisic acid, subsequently increasing the transcriptional levels of genes encoding sulfate transporters (Zou et al., 2021). The influence of jasmonic acid compounds on selenium uptake appears to be dependent on the concentration of selenium treatment. Under high selenium conditions, jasmonic acid compounds can reduce selenium uptake and accumulation in rice plants as a protective response. Additionally, they can also lower the selenium accumulation levels in tea leaves treated with high concentrations of sodium selenate (135 mg·m^-2^). Conversely, jasmonic acid compounds exhibit a significant promoting effect on the selenium content of tea leaves treated with low concentrations of sodium selenate (Dai et al., 2021). The impact of salicylic acid on selenium uptake varies depending on the crop. It has been shown to reduce selenium content in the roots and leaves of rice plants, while enhancing the uptake of organic selenium and sodium selenite in lettuce (Kowalska et al., 2020; Mostofa et al., 2020). In our investigation, genes associated with auxin, cytokinin, gibberellin, and brassinosteroid response elements were upregulated on day 3 of Se treatment (**Figures 4A–C, F**). Salicylic acid response element genes exhibited upregulation on day 12 of Se treatment (**Figure 4H**), while genes related to the jasmonic acid pathway showed downregulation post-Se treatment (**Figure 4G**). These results indicated that plant hormones also regulate the transport and accumulation of selenium.

### 4.4 Transcription factors involved in Se accumulation and transportation in foxtail millet

Transcription factors function as pivotal molecular switches, regulating growth and development in response to various conditions (Yuan, 2008). Specifically, bHLH transcription factors are involved in the uptake and distribution of iron in Arabidopsis (Riaz and Guerinot, 2021). Additionally, the transcription factors bZIP19 and bZIP23 act as central regulators in the zinc deficiency response, functioning as sensors for zinc by utilizing their Cys/His rich motif to bind Zn^2+^ ions (Lilay et al., 2021). Notably, previous investigations on tea plants have underscored the involvement of transcription factors, such as ERF, bHLH, and MYB, in the modulation of defense networks in response to SeO_3_^2−^ treatment, jasmonic acid, and ethylene exposure (Cao et al., 2018). Furthermore, the transcriptional level analysis has successfully established the association between selenium and the anthocyanin pathway through the participation of R2R3-MYB and bHLH in selenium metabolism (Pu et al., 2021). Moreover, the regulatory function of WRKY75 in tea roots has been identified in the accumulation of SeO_3_^2−^ (Zheng et al., 2023). In the analysis conducted using WGCNA, three hub genes were identified. Thus, in foxtail millet, the transportation of SeO_4_^2-^ from the leaf to the spike may be controlled by the transcription factors MYB, WRKY, and bHLH.

### 4.5 Selenium accumulation in foxtail millet enhanced antioxidant activity

Glutathione peroxidase and glutathione reductase are important enzymes and play a vital role in scavenging H_2_O_2_ and lipid peroxides to water and lipid alcohols, respectively (Hasanuzzaman and Fujita, 2011; Feng et al., 2013). GSH-Px is believed to be a key enzyme, which can be widely and robustly activated by Se in various plants exposed to several environmental stresses (Feng et al., 2013). In the presence of selenium, H_2_O_2_ (hydrogen peroxide) is primarily and majorly quenched by GSH-Px and then APX, CAT, and GR (Glutathione Reductase) eliminate the remnants of H_2_O_2._ Therefore, genes and proteins related to glutathione metabolism play important roles in assimilation and tolerance of Se in plants (Chauhan et al., 2020; Rao et al., 2021). Se induces positive responses in plant growth and development by elevating antioxidant defense systems, including CAT, GSH-Px, and SOD. It also fosters the accumulation of secondary metabolites, such as total phenols and flavonoids, fortifying membrane integrity and enhancing nutrient quality and crop productivity under diverse abiotic stresses (Hawrylak-Nowak et al., 2014). The genes encoding GST, GSS (glutathione synthetase), GSH-Px, and GR are notably increased in tea plants treated with selenite (Cao et al., 2018). Remarkably, the expression of glutathione metabolism related genes and proteins were highly induced even 3 days after treatments of selenate in this study. The genes encoding GST, GSH-Px, APX, MDHAR, and SOD exhibited significant upregulation post-Se treatment in this study (**Figure 5**), indicating their potential role in enhancing Se accumulation in foxtail millet.

### 4.6 Conclusion

As the duration of Se treatment on leaves increased, Se nutrients exhibited sequential transport and accumulation along the leaf-stem-ear axis. RNA-Seq analysis unveiled the transcriptional mechanisms of Se treatment, highlighting key genes involved in selenium transport following foliar application. Upregulation was observed in the transcription levels of sulfate transporters, phosphate transporters, nitrate transporters, antioxidant enzymes, transcription factors, and enzymes associated with plant hormone synthesis after Se treatment. The accumulation of Na_2_SeO_4_ in foxtail millet spikes correlated with the upregulation of transcription factors ABCC13, PHT1.3, SiNRT2.1, GSTs, MYB, WRKY, and bHLH. Notably, Na_2_SeO_4_, a key player in Se accumulation, significantly induced the expression of SiSULTR1.2b and SiSULTR3.1a. These findings form a foundational understanding of Se accumulation and transportation in foxtail millet.

## Data availability statement

The data presented in the study are deposited in the NCBI SRA database, accession number PRJNA1051663.

## Author contributions

YW: Writing – original draft. LC: Writing – original draft. ZZ: Data curation, Writing – review & editing. MA: Data curation, Writing – review & editing. SZ: Data curation, Writing – review & editing. JZ: Formal Analysis, Writing – review & editing. SD: Conceptualization, Writing – review & editing. XY: Conceptualization, Writing – review & editing. MY: Conceptualization, Writing – review & editing.
