# Supplementary material for: Transcriptome and co-expression network revealed molecular mechanism underlying selenium response of foxtail millet (Setaria italica)
Source: Front Plant Sci. 2024 Mar 11;15:1355518. doi: 10.3389/fpls.2024.1355518 (PMC10962390; doi:10.3389/fpls.2024.1355518)
Supplement: Supplementary file 1 [file DataSheet_1.pdf]

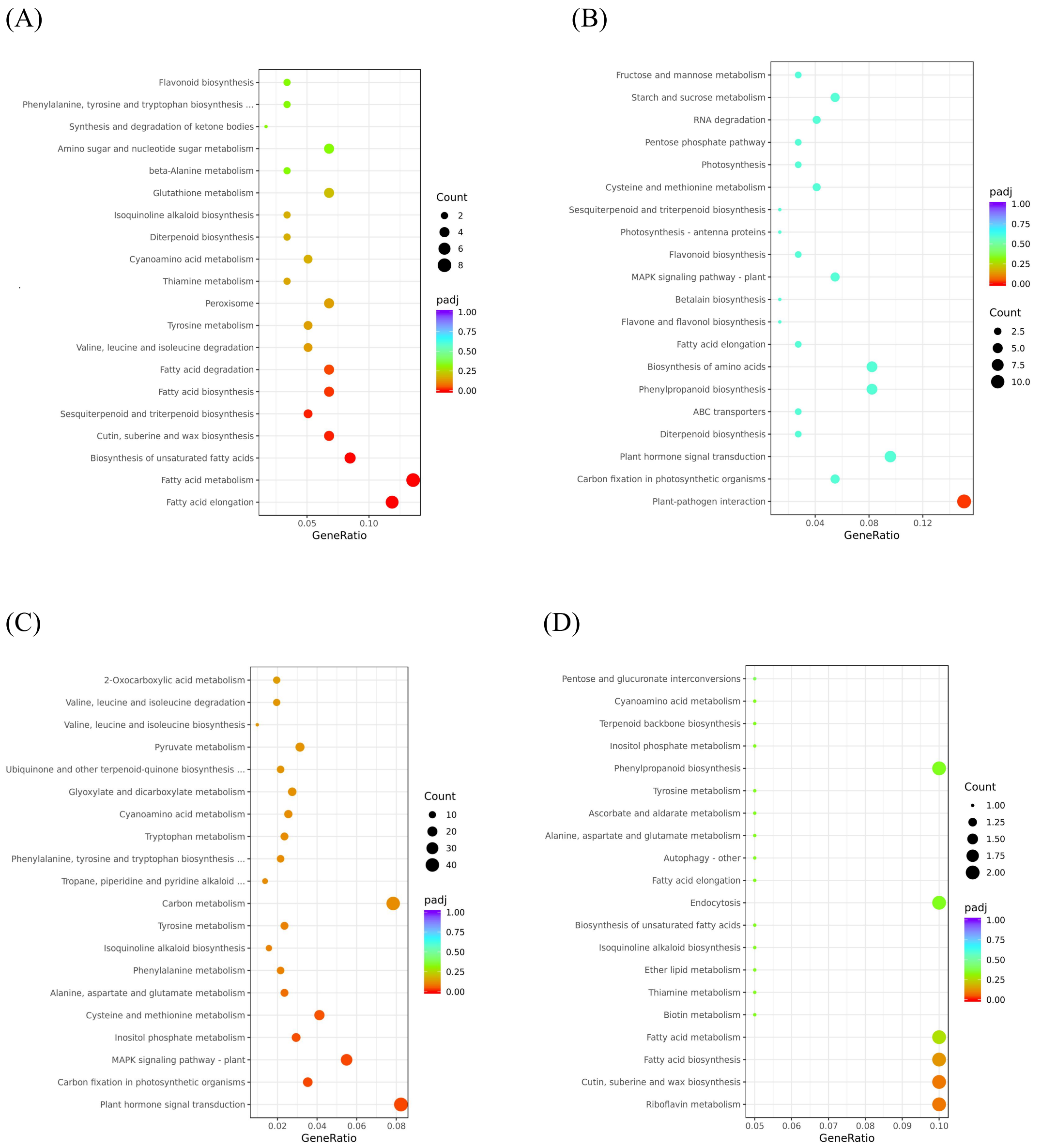
(A)
Flavonoid biosynthesis
Phenylalanine, tyrosine and tryptophan biosynthesis ...
Synthesis and degradation of ketone bodies
Amino sugar and nucleotide sugar metabolism
beta-Alanine metabolism
Glutathione metabolism
Isoquinoline alkaloid biosynthesis
Diterpenoid biosynthesis
Cyanoamino acid metabolism
Thiamine metabolism
Peroxisome
Tyrosine metabolism
Valine, leucine and isoleucine degradation
Fatty acid degradation
Fatty acid biosynthesis
Sesquiterpenoid and triterpenoid biosynthesis
Cutin, suberine and wax biosynthesis
Biosynthesis of unsaturated fatty acids
Fatty acid metabolism
Fatty acid elongation
0.05
0.10
GeneRatio
Count
2
4
6
8
padj
1.00
0.75
0.50
0.25
0.00
(B)
Fructose and mannose metabolism
Starch and sucrose metabolism
RNA degradation
Pentose phosphate pathway
Photosynthesis
Cysteine and methionine metabolism
Sesquiterpenoid and triterpenoid biosynthesis
Photosynthesis - antenna proteins
Flavonoid biosynthesis
MAPK signaling pathway - plant
Betalain biosynthesis
Flavone and flavonol biosynthesis
Fatty acid elongation
Biosynthesis of amino acids
Phenylpropanoid biosynthesis
ABC transporters
Diterpenoid biosynthesis
Plant hormone signal transduction
Carbon fixation in photosynthetic organisms
Plant-pathogen interaction
0.04
0.08
0.12
GeneRatio
padj
1.00
0.75
0.50
0.25
0.00
Count
2.5
5.0
7.5
10.0
(C)
2-Oxocarboxylic acid metabolism
Valine, leucine and isoleucine degradation
Valine, leucine and isoleucine biosynthesis
Pyruvate metabolism
Ubiquinone and other terpenoid-quinone biosynthesis ...
Glyoxylate and dicarboxylate metabolism
Cyanoamino acid metabolism
Tryptophan metabolism
Phenylalanine, tyrosine and tryptophan biosynthesis ...
Tropane, piperidine and pyridine alkaloid ...
Carbon metabolism
Tyrosine metabolism
Isoquinoline alkaloid biosynthesis
Phenylalanine metabolism
Alanine, aspartate and glutamate metabolism
Cysteine and methionine metabolism
Inositol phosphate metabolism
MAPK signaling pathway - plant
Carbon fixation in photosynthetic organisms
Plant hormone signal transduction
0.02
0.04
0.06
0.08
GeneRatio
Count
10
20
30
40
padj
1.00
0.75
0.50
0.25
0.00
(D)
Pentose and glucuronate interconversions
Cyanoamino acid metabolism
Terpenoid backbone biosynthesis
Inositol phosphate metabolism
Phenylpropanoid biosynthesis
Tyrosine metabolism
Ascorbate and aldarate metabolism
Alanine, aspartate and glutamate metabolism
Autophagy - other
Fatty acid elongation
Endocytosis
Biosynthesis of unsaturated fatty acids
Isoquinoline alkaloid biosynthesis
Ether lipid metabolism
Thiamine metabolism
Biotin metabolism
Fatty acid metabolism
Fatty acid biosynthesis
Cutin, suberine and wax biosynthesis
Riboflavin metabolism
0.05
0.06
0.07
0.08
0.09
0.10
GeneRatio
Count
1.00
1.25
1.50
1.75
2.00
padj
1.00
0.75
0.50
0.25
0.00

(E)

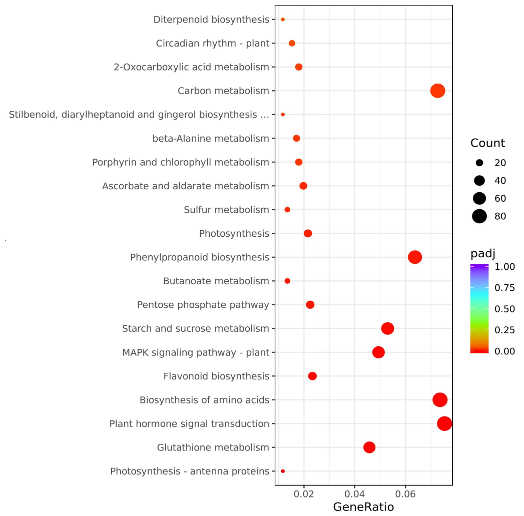

(F)

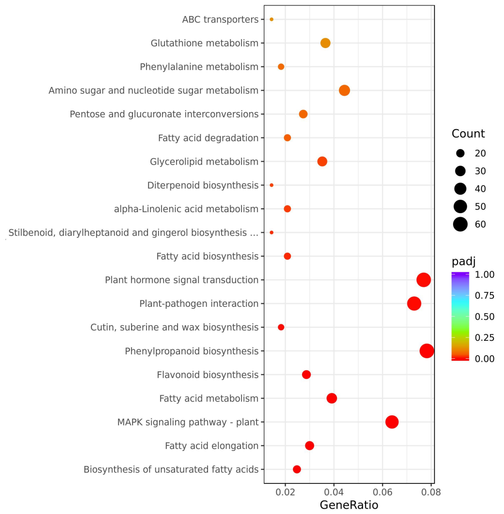

(G)

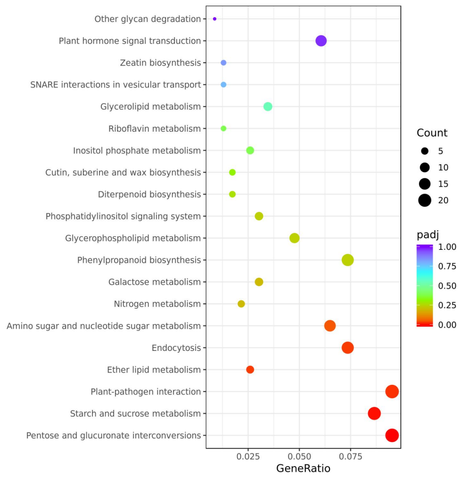

Supplementary Figure 1 20 top KEGG pathways enriched in differentially expressed genes (DEGs) among 7 comparisons, including Se3_v_CK3 (A), Se5_v_CK5 (B), Se7_v_CK7 (C), Se12_v_CK12 (D), Se5_v_Se3 (E), Se7_v_Se5 (F), Se7_v_Se12 (G).
